# A Study on the Presence of Osteopontin and α3β1 Integrin in the Endometrium of Diabetic Rats at the Time of Embryo Implantation

**Published:** 2020

**Authors:** Yasaman Zarrin, Abbas Bakhteyari, Parvaneh Nikpour, Fatemeh Sadat Mostafavi, Nahid Eskandari, Mohammad Matinfar, Roshanak Aboutorabi

**Affiliations:** 1- Isfahan Medical Student Research Center, Faculty of Medicine, Isfahan University of Medical Sciences, Isfahan, Iran; 2- Department of Anatomical Sciences, Faculty of Medicine, Isfahan University of Medical Sciences, Isfahan, Iran; 3- Department of Genetics and Molecular Biology, Faculty of Medicine, Isfahan University of Medical Sciences, Isfahan, Iran; 4- Child Growth and Development Research Center, Research Institute for Primordial Prevention of Non-Communicable Diseases, Isfahan University of Medical Sciences, Isfahan, Iran; 5- Department of Immunology, Faculty of Medicine, Isfahan University of Medical Sciences, Isfahan, Iran; 6- Department of Internal Medicine, Faculty of Medicine, Isfahan University of Medical Sciences, Isfahan, Iran

**Keywords:** Diabetes mellitus, Endometrium, Osteopontin, α3β1 integrin

## Abstract

**Background:**

Embryo implantation is a critical and multifactorial phenomenon which can be affected by any alteration in molecular micro construction of endometrium. The aim of the current study was to evaluate the effects of diabetes on osteopontin (OPN) and α3β1 integrin proteins level at the time of endometrial receptivity.

**Methods:**

Twenty-eight female rats were divided into control, diabetic, pioglitazone-treated and metformin-treated groups. Western blot was performed to determine the OPN and α3β1 integrin proteins in rats’ endometrium at the time of implantation. Data were analyzed by analysis of variance (ANOVA) and p<0.05 was considered statistically significant.

**Results:**

OPN increased significantly in the diabetic group in comparison with control (p<0.001), metformin-treated (p=0.008) and pioglitazone-treated groups (p< 0.001). Furthermore, α3β1 integrin protein level in diabetic group had a significant difference in comparison with that of the control (p<0.001), metformin-treated (p= 0.026) and pioglitazone-treated groups (p<0.001).

**Conclusion:**

OPN and α3β1 integrin proteins are involved in embryo implantation and their changes in diabetic condition can affect fertility. Treatment with pioglitazone and metformin improved the level of OPN and α3β1 integrin proteins while pioglitazone was more effective.

## Introduction

Diabetes mellitus (DM) and its complications are going to be a serious health issue around the world (1) with a high prevalence in Asia (2). Type 2 diabetes mellitus (T2DM) accounts for more than 90% of all cases of DM and its prevalence is rapidly increasing universally (2). DM, as a metabolic disorder, is a chronic condition which causes serious complications in various organs such as nephropathy, retinopathy, neuropathy, stroke and myocardial infarction (3). In addition to the complications mentioned above, DM also affects the male and female reproductive system and it can reduce the rate of fertility and increase reproductive problems (4). The prevalence of infertility has been reported differently all over the world, but it involves 8–12% of all couples in the world (5).

Infertility is a complicated and multifactorial disorder and nearly 15% to 30% of couples are diagnosed with unexplained infertility (6). There are several potential reasons for unexplained infertility and failure in implantation is one of the most important causes for the problem (7). In women with DM, the rate of pregnancy is lower than that of healthy and non-diabetic women (4). According to literature review, fertility could be severely affected by diabetes mellitus from embryo implantation phase until the birth (8, 9).

Blastocyst implantation is a process in which the trophoblast cooperates with endometrial epithelial cells (10). Implantation begins with a weak and feeble connection between the embryo and endometrium, which leads to binding and penetration into the endometrial tissue (11). Diabetes mellitus can alter the molecular pathways in the female reproductive system, therefore leading to the disruption of implantation process and subsequently loss of the fetus (12). The pattern of integrin proteins expression has a significant relationship with fertility and implantation process (13, 14).

The rate of spontaneous abortion increases in women with uncontrolled hyperglycemia (15, 16). Implantation of the embryo in the uterus requires the cross talk and concurrency of several molecules. In this phenomenon, different adherent molecules, such as integrins and osteopontin are involved (17).

These adhesion molecules are regularly expressed at the time of endometrial receptivity which ultimately could lead to a successful implantation (18, 19). Integrins are a member of heterodimeric transmembrane glycoproteins. Their bio-construction is formed by non-covalent connection of α and β subunits. Each α or β subunit consists of an extracellular domain, an intracellular domain and a transmembrane portion (20). These adhesion molecules facilitate cell attachment and connect extracellular matrix to specific ligands such as collagen IV, fibronectin, laminin and osteopontin (21). Integrin α3β1 is continuously expressed during a normal menstrual period and has the role as a receptor for collagen and osteopontin in the extracellular matrix (20).

Osteopontin, or secreted phosphoprotein 1 (SPP1) is an acidic, single chain, phosphorylated glycoprotein which is produced by various cells such as mesenchymal stem cells (22), epithelial cells (23), T cells, macrophages and dendritic cells (24). The OPN molecule is located in extracellular matrix and acts as a receptor for integrins and influences various functions in endometrial epithelial cells (24). The level of OPN protein is higher in diabetic patients than in healthy people (25). Gong et al. also demonstrated that OPN levels are increased during the early stages of hyperglycemia (26). In the uterus and during implantation, OPN is expressed and increases dramatically in the secretion phase (27, 28). The expression of OPN at this stage reflects the role of this molecule in the implantation process, which has attracted the attention of researchers (28, 29). Expression of integrin is affected by diabetes (30).

There are different blood glucose regulatory drugs and among them, two groups *i.e*. biguanide (Metformin) and thiazolidinedione (Pioglitazone) have been evaluated in this study. Metformin is the most frequently prescribed drug for diabetes treatment (31). Reduction of hepatic glucose production has been suggested as the primary function of this drug. Several mechanisms are involved in this procedure such as changes in enzymes activities or decrease in hepatic gluconeogenic substrates absorption (32).

Pioglitazone, a thiazolidinedione derivative, is an insulin-sensitizing agent regulator for the treatment of T2DM (33). Pioglitazone is a peroxisome proliferator-activated receptor gamma (PPAR-γ) agonist which can reduce insulin resistance in liver, muscle and adipose tissue (34) and improve glucose and lipid metabolism (33).

Due to the high prevalence of diabetes mellitus and its effects on fertility, especially at the molecular level, the aim of our study was to examine the impression of diabetes on OPN and α3β1 integrin proteins values in endometrium at the time of embryo implantation in diabetic rat models treated with either metformin or pioglitazone.

## Methods

This interventional study on diabetes mellitus was performed according to the certificate from Ethics Committee at Isfahan University of Medical Sciences, Isfahan, Iran (IR.MUI.REC.1396.1. 189). The experiment was conducted on twenty-eight adult virgin female Wistar rats aged seven to eight weeks with weight of 175–225 *gr* purchased from Pasteur Institute of Iran. Animals were bred in normal conditions of 40–70% humidity, temperature of 21±4*°C* and on a 12 *hr* light/dark cycle.

### Induction of diabetes:

In order to induce experimental DM, rats were fasted overnight. In the next morning, nicotinamide (NA, Sigma-Aldrich, Germany) and streptozotocin (STZ, Sigma-Aldrich, Germany) were prepared in normal saline and intraperitoneally injected. First, nicotinamide (200–230 *mg/kg*) was injected, then after 15 *min*, it was followed by 60 *mg/kg* of streptozotocin injection. In order to measure fasting blood sugar, three days after intraperitoneal injection of NA and STZ, blood sampling was done by glucometer (HemoCue Glucose 201+, Sweden) and fasting blood sugar (FBS) was recorded in all rats. Rats with FBS in range of higher than 250 *mg/dl* were considered as diabetic models (35).

### Study design and sampling:

**Diagram 1.**
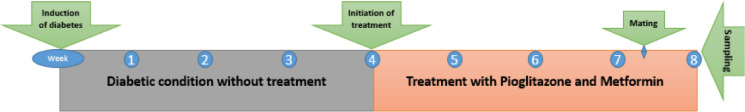
The study was designed from the induction of diabetes till tissue sampling. This study lasted 8 weeks, 4 weeks in diabetic condition without treatment and the other 4 weeks, treatments with pioglitazone and metformin were applied

Twenty-eight rats were randomly divided in four groups as follows: control group, STZ+NA induced diabetic model group without any treatment (FBS ≥250 *mg/dl*), diabetic group which received pioglitazone (20 *mg/kg/day* by orogastric gavage) and the last group was diabetic rats which received metformin (100 *mg/kg/day* by orogastric gavage). The rats were kept in diabetic condition for 4 weeks and then during the other next 4 weeks, metformin and pioglitazone treatment initiated. FBS was monitored every 4 days using a glucometer (HemoCue Glucose 201+, Sweden) and glucose reagent strips (ACCU-CHEK Active, Germany). In order to determine implantation time, female rats were mated with male rates. Mating of animals was conducted by placing each two female rats (16 weeks of age) with one male rat. In the next morning, observation of vaginal plugs or obtaining vaginal smears was the indication for the first gestational day. Ninety six *hr* (4 days) after observation of vaginal plugs or sperm in vaginal smears was the implantation time. Four weeks after treatment with metformin or pioglitazone, at the time of blastocyst implantation which was at the end of 8th week, rats in all groups were fasted overnight and then sacrificed by intraperitoneal injection of ketamine hydrochloride (50 *mg/kg*) and xylazine hydrochlo ride (7 *mg/kg*) (Diagram 1). Their uterine horns were dissected and endometrium was removed in a sterile condition. The endometrial samples were washed with Hanks’ balanced salt solution, then chopped in several fragments and kept in the sterile micro tubes.

### Western blot analysis:

Uterine slices, previously frozen at −80°*C*, were incubated and lysed in radioimmunoprecipitation assay buffer (RIPA buffer) (Cytomatingen, Iran). The protein concentration was quantified using Bradford assay. Sodium dodecyl sulfate-polyacrylamide gel electrophoresis (SDS-PAGE) using a 10% polyacrylamide gel was performed and the proteins were transferred to nitrocellulose membranes (Bio-Rad, Hercules, CA, USA). The membranes were blotted with rabbit anti- OPN (cat. no. ab8448; Abcam) and rabbit anti α3β1 integrin (cat no. bs-1057R, bioss) primary antibodies at a dilution of 1:250 and incubated overnight at 4°*C*. Following incubation, the membranes were washed three times with phosphate-buffered saline containing 0.05% Tween 20 and then incubated with mouse anti-rabbit IgG – HRP secondary antibody (cat. nos. P1308 and P1309; Applygen Technologies) at a dilution of 1: 1,000 at room temperature for 90 *min*. The blots were visualized with Clarity western ECL substrate (cat. no. Bio-Rad, 170–5060, USA). The densitometry of bands was performed using ImageJ software (Version 1.8, National Institutes of Health, USA) and β-actin (cat. no. ab8226; Abcam) was used as the reference protein to validate the amount of protein loaded onto the gel.

### Statistical analysis:

Statistical analysis was performed using Statistical Package for the Social Sciences (SPSS) software, version 20.0 (SPSS Inc., USA). All experiments were performed at least three times and final results were expressed as means±standard error of mean (SEM). Analysis of variance (ANOVA) followed by Tukey test was utilized to detect statistical significance which was considered as p<0.05.

## Results

### The protein level of OPN:

**Figure 1.**
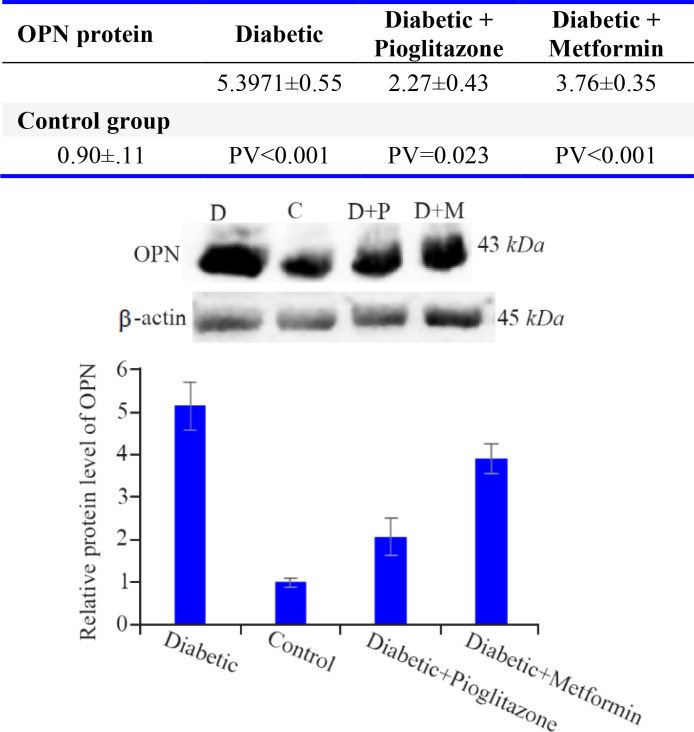
Comparison of OPN protein levels in the rat endometrium at the time of embryo implantation. All values were presented as mean±SEM. Error bars represent standard error of mean. p<0.05 was considered statistically significant. Data analysis reveals that significant differences were observed between all four groups. In western blot image, D=diabetic control, C=control group, D+P=diabetic rats treated with pioglitazone and D+M=diabetic rats treated with metformin

According to figure 1, the protein level of OPN was increased significantly in diabetic group in comparison with control (p<0.001), metformin-treated (p=0.008) and pioglitazone-treated (p<0.001) groups. Furthermore, there was a significant difference between control group compared with metformin-treated (p<0.001) and pioglitazone-treated (p=0.023) groups. The level of OPN protein in pioglitazone-treated group was significantly lower than metformintreated group (p=0.014).

### The protein level of α3β1:

**Figure 2.**
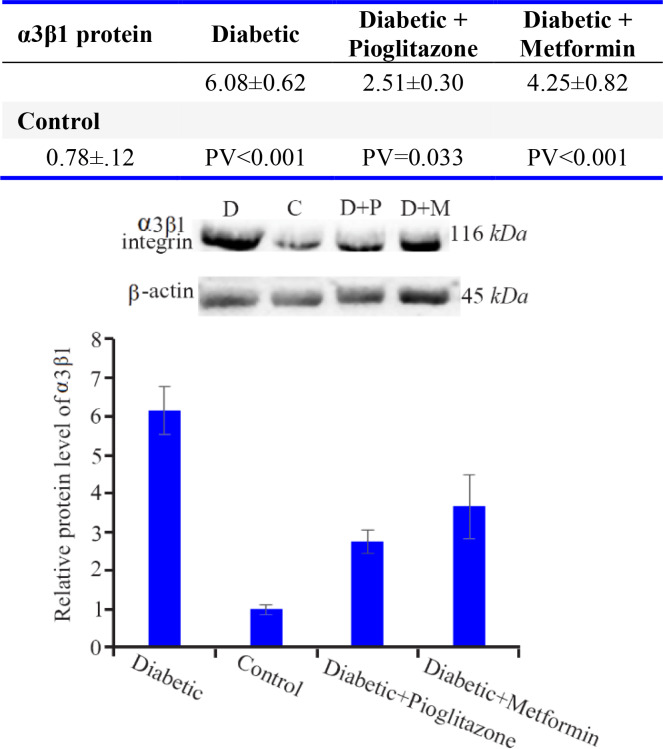
Comparison of α3β1 integrin protein levels in rat endometrium at the time of embryo implantation. All values were presented as mean±SEM. Error bars represent standard error of mean. p<0.05 was considered statistically significant. Data analysis reveals that significant differences were observed between all four groups. In western blot image, D=diabetic control, C=control group, D+P=diabetic rats treated with pioglitazone and D+M=diabetic rats treated with metformin

As figure 2 reveals, the mean of α3β1 integrin protein level in the diabetic group showed a significant difference in comparison with control (p<0.001), metformin-treated (p= 0.026) and pioglitazone-treated (p<0.001) groups. There was also a significant difference between control group compared with metformin-treated (p<0.001) and pioglitazone-treated (p=0.033) groups. The level of α3β1 integrin protein was significantly lower in pioglitazone-treated than metformin-treated group (p=0.033).

## Discussion

In 2013, International Diabetes Federation (IDF) reported that nearly 382 million people were suffering from diabetes mellitus worldwide, and according to their prediction, it may reach 600 million in 2030 (36). About 90% of cases with T2DM are diagnosed in the ages less than 30 years old. With onset of diabetes at a younger age, it ought to be considered that its beginning is in the middle range of woman’s reproductive years (37, 38). Therefore, the complications and side effects of DM could be observed in women at their golden time of fertility. As previously reported, fertility rate is lower in women with DM than in healthy women, and DM could be assumed as one of the subfertility causes (39, 40).

DM could alter the value of OPN and α3β1 integrin proteins (26, 30, 41, 42). In the early stage of diabetic nephropathy in human, α3β1 integrin gene expression significantly increases in kidney (Podocytes) (43). Setty et al. reported the increased expression of α3 protein in meningeal cells cultured in high-glucose media (44, 45). The protein level of OPN, in the vitreous body of patients with diabetic retinopathy, is up-regulated compared to non-diabetic individuals (46, 47). In rat models of diabetic nephropathy, OPN expression is obviously elevated in the aorta and renal cortex (48, 49). Therefore, according to literature review, increased OPN levels could be considered as one of the primary factors of the complicated malfunctions in affected organs.

At the secretory phase of endometrium, α3β1 protein expression is up-regulated and this alteration could be due to the hormonal cycle (50). DM affects the integrins synthesis and integrins functions against extracellular matrix. So, it could be assumed as the cause of abnormal and firmer attachment in the affected epithelium (51).

Qi et al. demonstrated that OPN mRNA and OPN protein are up-regulated on day 4th of pregnancy, suggesting that OPN may play a role in the blastocyst implantation (52). OPN protein level was reported to be raised in the endometrial epithelium in the mid-secretory phase of menstrual cycle (12, 53).

In the present study, DM affected the expression of OPN and integrin α3β1 proteins and up-regulated their expression levels in the endometrium at the time of embryo implantation. With consideration of these facts about OPN and α3β1 integrin and their specified roles in implantation, the increased value of these proteins in DM may lead to more tight junctions between endometrial epithelial cells at the time of embryo implantation. These disturbances in the amounts of these proteins could lead to unsuccessful endometrial receptivity.

No similar study was found for measuring the effects of diabetes on OPN and α3β1 integrin proteins in the endometrium. But in our previous study, diabetes was found to increase the gene expression of OPN in the endometrium, which was in line with the results of the present study (12).

In our present study, the effects of two common hypoglycemic drugs *i.e*. metformin and pioglitazone, were also evaluated in order to compare these two drugs’ impressions on OPN and α3β1 integrin proteins expression. The results demonstrated that both drugs could significantly reduce the levels of OPN and α3β1 integrin proteins compared with diabetic group. Notably, pioglitazone was more effective than metformin and reduced the OPN and α3β1 integrin protein levels more than metformin.

Pioglitazone improves glucose plasma levels in patients with T2DM by increasing insulin sensitivity in different cells (54). In the present study, pioglitazone showed a more prominent effect on controlling the protein levels of OPN and integrin α3β1. Better efficacy of pioglitazone than metformin may be due to the interaction of pioglitazone with the nuclear receptor called peroxisome proliferator-activated receptor gamma (PPARγ) (One of regulators of genes expression) which affects DNA transcription in endometrial tissue at the time of embryo implantation (12).

## Conclusion

Diabetes mellitus significantly increased the protein levels of OPN and α3β1 integrin in the rat endometrium at the time of embryo implantation. And it seems untreated diabetes could be potentially assumed as one of the elements in embryo implantation failure in assisted and natural reproduction. Treatment with pioglitazone and metformin improved the level of OPN and α3β1 integrin proteins while interestingly pioglitazone was more effective.
